# The Identification of Immune-Related Biomarkers for Osteoarthritis Immunotherapy Based on Single-Cell RNA Sequencing Analysis

**DOI:** 10.1155/2023/5574636

**Published:** 2023-03-14

**Authors:** Zhe Tan, Rong Chen, Hanyu Lin, Hong Wang

**Affiliations:** Orthopedics Department, Yaan Hospital of Traditional Chinese Medicine, Yaan, China

## Abstract

Osteoarthritis (OA) is a chronic musculoskeletal disease affecting approximately 500 million people worldwide. Globally, OA is one of the most common and leading causes of disability. Several genetic factors are involved in OA, including inherited genes, genetic susceptibility, and genetic predisposition. As the pathogenesis of OA is unknown, there are almost no effective treatments available to prevent the onset or progression of the disease. In recent years, many researchers focused on bioinformatics analysis to explore new biomarkers for the diagnosis, treatment, and prognosis of human diseases. In this work, we obtain the traditional RNA sequencing data of OA patients from the GEO database. By performing the differentially expressed analysis, we successfully obtain the genes that are closely associated with the OA. In addition, the Venn diagram was applied to evaluate the genes that are involved in OA and immune-related genes. The protein-protein interaction analysis was further conducted to explore the hub genes. The single-cell RNA sequencing analysis was used to evaluate the expression distribution of the MMP, VEGFA, SPI1, and IRF8 in synovial tissues of patients with osteoarthritis. Finally, the GSVA enrichment analysis discovered the potential pathways involved in OA patients. Our analysis provides a new direction for the exploration of the process of OA patients. In addition, VEGFA may be considered a promising biomarker in OA.

## 1. Introduction

The cartilage in synovial joints is a transparent tissue that covers the bony surface, making it easier for the joint to slide and friction to be reduced [1]. Synovial fluid and subchondral bone nourish this tissue, and its physical properties include resistance to pressure and compressive forces [2]. The only type of cell present in cartilage is the chondrocyte, which accounts for 1–5% of the total cartilage mass [3]. The chondrocytes are grounded in collagen and proteoglycans, which are considered an amorphous extracellular matrix. During compression, cartilage is protected by proteoglycans, which provide tension to the tissue [4]. Among all joint diseases, osteoarthritis (OA) is one of the most common and leading causes of disability in the world. Approximately 18% of women and 9.6% of men over the age of 60 have symptoms of OA, and a quarter is unable to carry out daily activities because of it [5]. The number of people suffering from OA is expected to rise to 130 million by 2050, representing a major social issue [6]. OA is primarily caused by genetic factors such as inherited genes, genetic susceptibility, and genetic predisposition [7]. There are almost no treatments available to prevent the onset or progression of OA because no clear pathogenesis has been identified. As opposed to earlier paradigms, OA is now recognized as a low-grade inflammatory disease that affects the entire joint, such as progressive destruction of articular cartilage, synovitis, subchondral bone remodeling, osteophyte formation, and meniscal and ligament changes [8]. It is becoming increasingly evident that inflammation (especially synovitis) plays an important role in OA as well as mechanical load.

The search for biomarkers for OA prevention, diagnosis, and disease progression has been a major focus of many researchers in recent years. It has been shown that the combined detection of serum chondroitin sulfate epitope 846 and cartilage oligomeric matrix protein is an effective method for diagnosing and monitoring the progression of osteoarthritis [9]. The collagen II C-terminal peptide concentration was higher in synovial fluid in patients with early OA compared to healthy controls [10]. In urine samples from patients with knee OA, metabolite levels may be capable of predicting the progression of the disease. In addition, C-reactive protein is associated with knee OA occurrence and progression [11]. A patient's susceptibility to OA progression can be distinguished by acids such as glycolic acid, hippuric acid, and fenugreek. Due to the small sample sizes of the above studies, these results are limited [12]. At the present time, there are very few biomarkers available for clinical use. Therefore, it is urgent to explore more effective biomarkers to better prevent, diagnose, and treat OA.

In recent years, with the quick development of bioinformatics analysis, in silico analysis has been widely taken into consideration for the analysis of the etiology, prevention, and prognostic prediction [13–15]. In this work, the aim is to explore the genes that play a key role in the occurrence of OA. In addition, the potential enrichment analysis was also used to explore the enriched pathways that are closely associated with OA. Further, the immune cell infiltration analysis was performed to further explore the correlation between OA and immune-related cells.

## 2. Methods

### 2.1. The Downloaded Dataset of OA Patients

Based on the GEO database, we downloaded the gene expression profile of GSE98918. A total of 24 synovial tissue samples are included in GSE98918, including 12 samples from normal joints and 12 samples from joints with OA. Data analysis was performed using R and Bioconductor software packages. The “sva” package is used to eliminate batch effects and normalize data.

### 2.2. The Screening of the Differentially Expressed Genes (DEGs) between Normal Synovial Tissue and Synovial Tissue Samples with OA

Through comparison of expression values in synovial tissues from normal joints and DEGs from OA joints, the LIMMA package in Bioconductor was used to identify DEGs and OA joints. *P* value <0.05 and |log_2_FC| > 1 were used as selection criteria. The pheatmap package is used to draw heatmaps of DEGs in R software.

### 2.3. The Exploration of the Potential Pathways That Are Closely Associated with the Differentially Expressed Genes

Functional enrichment was used to further confirm the potential functions of the potential targets. GO is widely used for annotating genes with functions, particularly molecular functions (MFs), biological pathways (BPs), and cellular components (CCs). In addition, analyzing gene function and related high-level genome function information using KEGG enrichment analysis is practical and useful. Analysis of the GO function of potential mRNAs and enrichment of KEGG pathways were performed using the ClusterProfiler package in R to better understand the oncogenic functions of target genes.

### 2.4. Protein-Protein Interaction Network Based on the Key Genes

In order to explore the potential correlation between the proteins encoded by key genes, we then constructed the PPI network. Using STRING, a gene PPI network was analyzed interactively. PPI networks were also analyzed and visualized using Cytoscape 3.8.2 when interactions with composite ratings exceeded 0.4.

### 2.5. Immune Cell Infiltration

CIBERSORT (https://cibersort.stanford.edu/) is an online analysis tool for estimating the abundance of many immune cell subtypes in mixed cell populations using gene expression data.

### 2.6. The Single-Cell RNA Sequencing Analysis in the Synovial Tissues of Patients with Osteoarthritis

In the single-cell RNA sequencing analysis, all the data were obtained from the GEO database (https://www.ncbi.nlm.nih.gov/geo). The Seurat package is used to generate objects and filter out cells with poor quality. Additionally, standard data preprocessing procedures are performed, resulting in the determination of gene number, cell number, and mitochondrial content. We filter the samples based on genes and cells containing fewer than 200 genes. Cells with at least three genes detected were retained, while cells with fewer than 500 or more than 7500 genes detected were discarded. Cells with high mitochondrial content (>20%) were also removed. The UMI counts were scaled with scale factor = 10,000. After log-transforming the data, we used the ScaleData function in Seurat to normalize each cell. As a result, we first identified some hypervariable genes in all samples, also known as magnet genes (genes that exhibit obvious differential expression between groups) which were used for grouping and cell identification. The marker gene of each cluster identifies each cluster. Generally, the marker gene is expressed characteristically in this type of cell and requires at least two other characteristic genes to describe it as a whole. Renaming of cell populations is carried out after all populations have been identified based on the annotation results.

### 2.7. Gene Set Variation Analysis (GSVA) of the Hub Genes

In this study, gene set enrichment was evaluated using GSVA, an unsupervised, nonparametric method. As a result of scoring the genes of interest and determining the biological function of the sample, changes at the gene level were transformed into changes at the pathway level in this study. Based on the molecular signatures database (version 7.0), gene sets were retrieved. In order to evaluate potential biological function changes, various samples were evaluated using the GSVA algorithm [13].

## 3. Results

### 3.1. A Total of 220 Genes Were Considered the DEGs and Show Some Key Pathways

**Figure 1:**
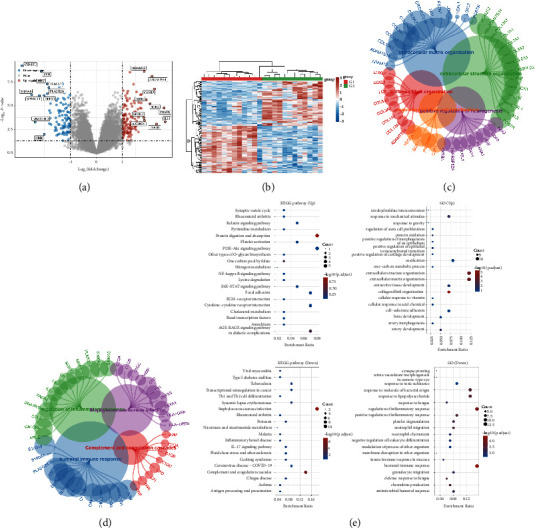
(a) The volcano map demonstrated the DEGs between OA patients and normal cohort. (b) The heatmap demonstrated the DEGs between OA cohort and normal people. (c) The GO enrichment analysis. (d) The KEGG enrichment analysis. (e) The pathway enrichment analysis based on the DEGs.

In the gene expression profile of GSE98918, a total of 220 genes were considered as the DEGs, which include 127 upregulated genes and 93 downregulated genes (Figures 1(a) and 1(b)). Subsequently, we also performed the GO enrichment analysis based on the DEGs. The results revealed that some pathways are closely associated with the upregulated genes, including extracellular matrix organization, extracellular structure organization, collagen catabolic process, regulation of stem cell proliferation, and positive regulation of morphogenesis of an epithelium. However, the downregulated genes are highly correlated with the pathways, such as humoral immune response, complemental and coagulation cascades, regulation of inflammatory response, and *Staphylococcus aureus* infection. In addition, the KEGG enrichment analysis demonstrated that the upregulated genes are closely associated with the synaptic vesicle cycle, relaxin signaling pathway, pyrimidine metabolism, protein digestion, and absorption. However, the downregulated genes involved in the DEGs are closely associated with the pathways, such as viral myocarditis, Th1 and Th2 cell differentiation, transcription misregulation in cancer, and the IL-17 signaling pathway (Figures 1(c)–1(e)).

### 3.2. Exploration of the Different Immune-Related Cells That Are Closely Associated with the DEGs

**Figure 2:**
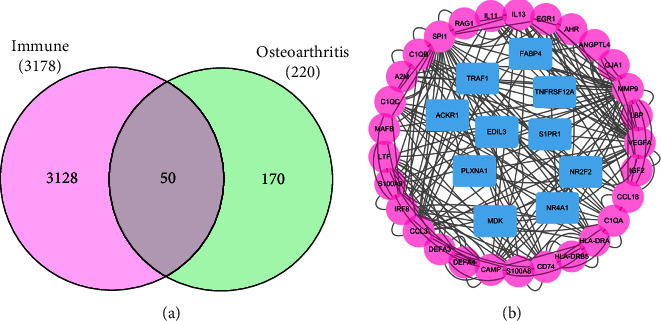
(a) The Venn diagram demonstrated the genes that are closely associated with DEGs and immune-related genes. (b) The PPI network based on the 50 key genes.

In order to explore the genes that are closely associated with the immune-related indexes, we downloaded the immune-related genes from the former studies. We finally obtained a total of 3178 immune-related genes. In addition, 50 of them are also involved in the differentially expressed genes in the OA cohort (Figure 2(a)). Subsequently, in order to further explore the hub immune-related genes that may play a key role in the OA cohort, we then constructed the PPI network. The results demonstrated that many genes show more than 20 interactive counts with other genes, such as MMP9 (36 interactive counts), VEGFA (32 interactive counts), SPI1 (28 interactive counts), IRF8 (20 interactive counts), and CAMP (20 interactive counts). Therefore, these five genes were considered as the hub immune-related genes involved in the OA cohort (Figure 2(b)).

### 3.3. The Single-Cell RNA Sequencing Analysis Revealed Different Cell Types Involved in the Synovial Tissues of Patients with Osteoarthritis

**Figure 3:**
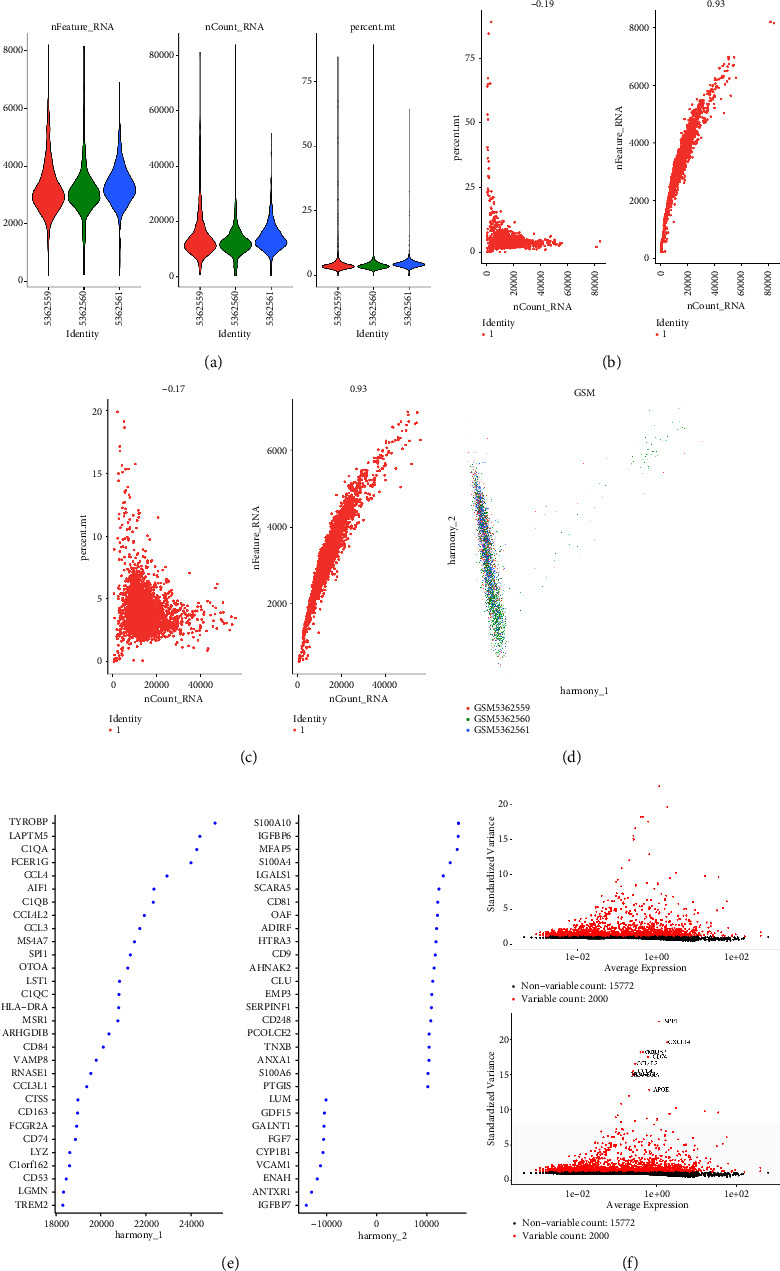
(a) Gene data detected in each cell, total number of molecules detected in the cell, and fraction of the mitochondrial genome in the cell. (b) Correlation between basic data before filtering. (c) Correlation between filtered base data. (d) After PCA, multiple PC groups with large differences can be used as anchor points. (e, f) The screening of the hypervariable genes.

**Figure 4:**
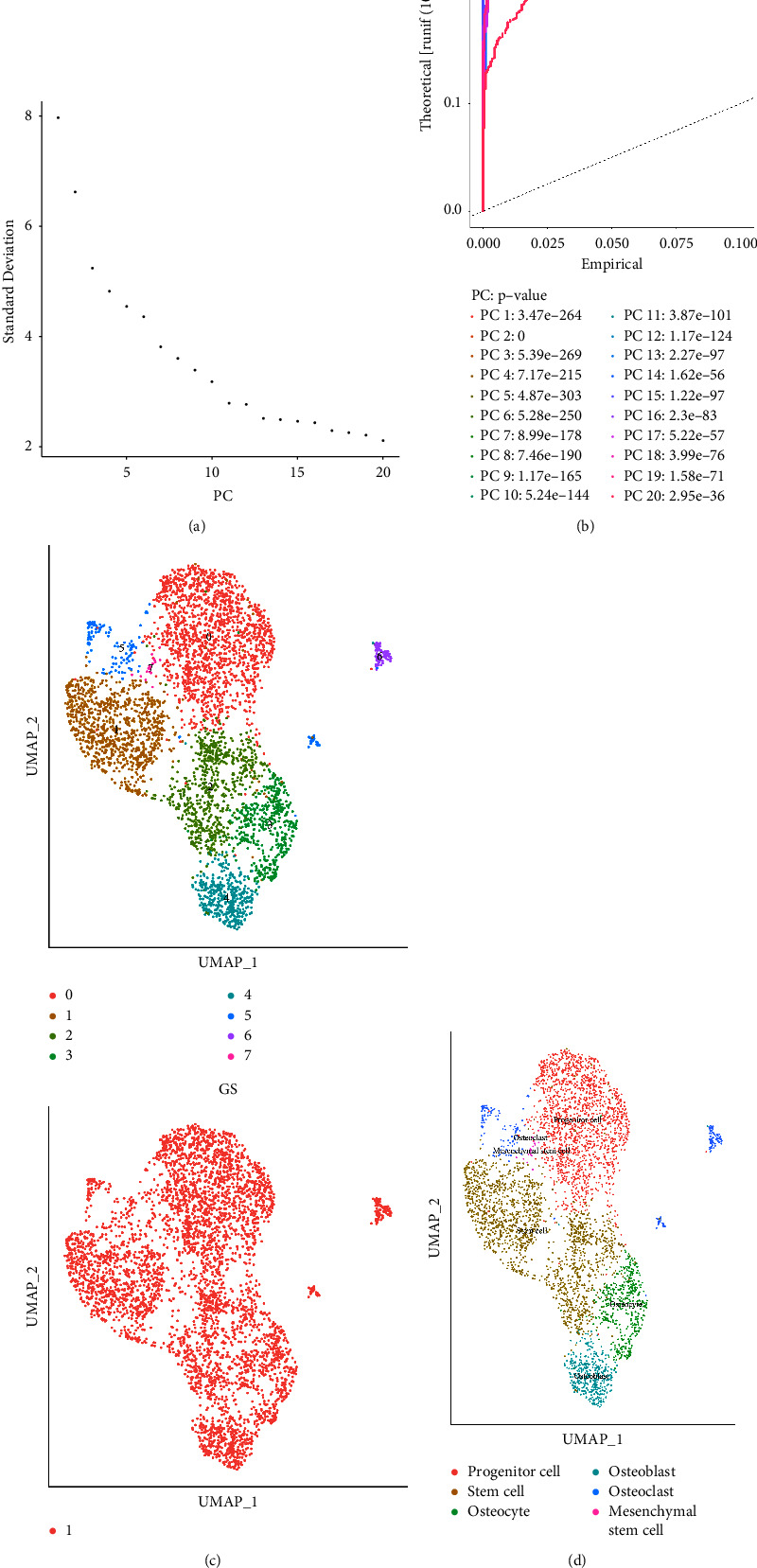
(a) The ElbowPlot function was used to evaluate PC. (b) Visualization results of the JackStrawPlot function for comparing the distribution of *P* values for each PC to the uniform distribution (dashed line). Significant PCs are shown above the dashed line with a significant *P* value. (c) Cluster results by UMAP method. (d) Proportional distribution of cells in the sample.

The single-cell RNA sequencing data of GSE176308 with the platform of GPL18573 were applied for further analysis, which consist of 3 samples of synovial tissues of the patients with osteoarthritis. Firstly, we performed the quality control of cells. The standard of RNA counts is set between 500 and 7500. In addition, cells with high mitochondrial content (>20%) were also removed (Figure 3(a)). Subsequently, a subset of features of hypervariable genes was used to perform batch removal analysis on the filtered data (Figures 3(b)–3(d)). Hypervariable genes are the genes that show the largest difference in expression from cell to cell (Figures 3(e) and 3(f)). We then transform highly variable genes into highly variable gene groups by using principal component analysis (Figures 4(a)–4(c)). After performing the cell annotation, we finally obtained a total of 6 types of cells in the synovial tissues of patients with osteoarthritis, which include progenitor cell, stem cell, osteocyte, osteoblast, osteoclast, and mesenchymal stem cell (Figure 4(d)). In addition, the proportional histogram demonstrated that most enriched cell type is the stem cell. In addition, the second enriched cell type is the progenitor cell. Furthermore, osteocytes, osteoblasts, and osteoclasts also account for the majority of the cells in the synovial tissues of patients with osteoarthritis.

### 3.4. The Expression Level of MMP9, VEGFA, SPI1, and IRF8 in Single-Cell RNA Sequencing Dataset

**Figure 5:**
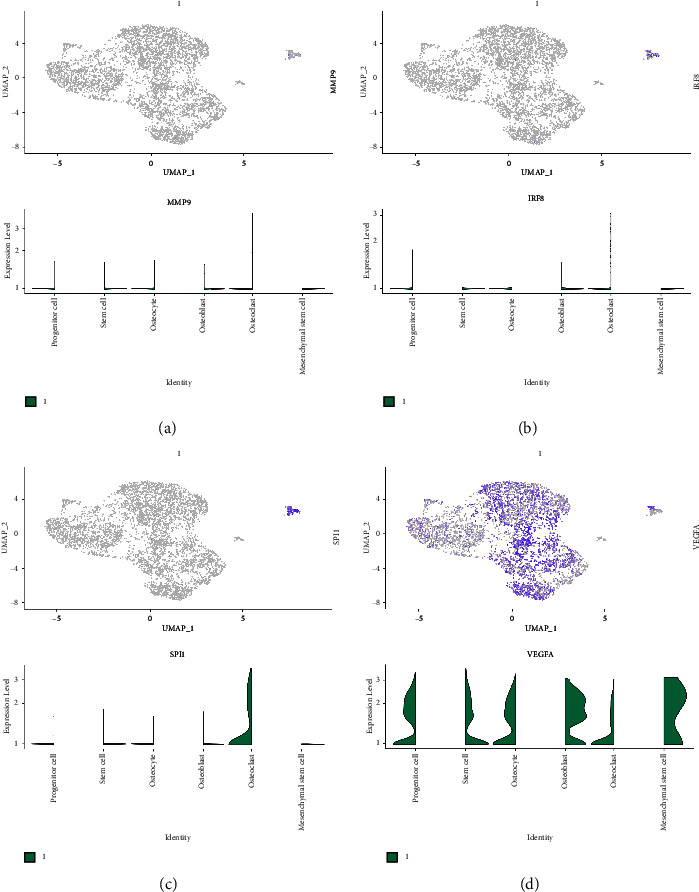
(a) The single-cell RNA sequencing analysis of MMP9. (b) The single-cell RNA sequencing analysis of IRF8. (c) The single-cell RNA sequencing analysis of SPI1. (d) The single-cell RNA sequencing analysis of VEGFA.

Based on the previous study, we then evaluate the expression distribution of the MMP, VEGFA, SPI1, and IRF8 in synovial tissues of patients with osteoarthritis. The results demonstrated that MMP9 is not especially expressed in the cells of synovial tissues (Figure 5(a)). For IRF8, the results demonstrated that the osteoclast is the most enriched cell in the synovial tissues (Figure 5(b)). In terms of SPI1, the most enriched cells in synovial tissues are also osteoclasts (Figure 5(c)). Finally, we evaluate the expression of VEGFA in synovial tissues. The single-cell RNA sequencing analysis demonstrated that progenitor cells, stem cells, osteocytes, osteoblasts, osteoclasts, and mesenchymal stem cells are closely associated with the VEGFA (Figure 5(d)).

### 3.5. Exploration of the Potential Pathways That Are Closely Associated with MMP9, VEGFA, SPI1, and IRF8

**Figure 6:**
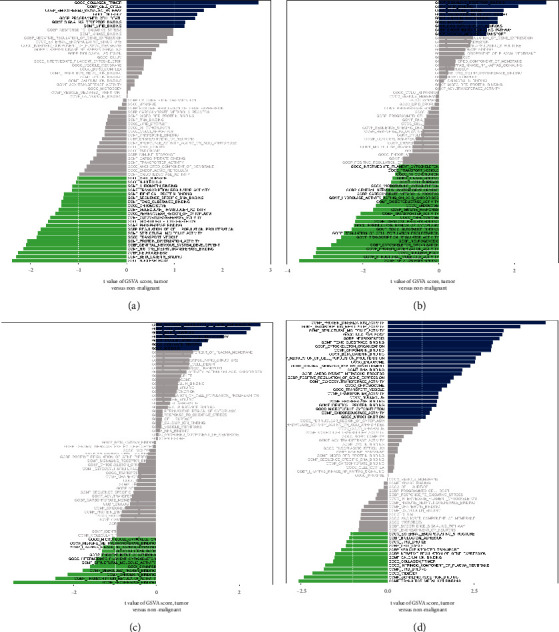
(a) GSVA of IRF8. (b) GSVA of MMP9. (c) GSVA of SPI1. (d) GSVA of VEGFA.

Subsequently, in order to further explore the potential pathways that are closely associated with the four key immune-related genes that are also highly correlated with the OA, we then performed GSVA to explore the potential pathways. The high expression level of IRF8 is associated with collagen trimer, cell cycle, smoothened signaling pathway, midbody, programmed cell death, and signaling receptor binding, while the downregulation of IRF8 is associated with the nuclear body, beta-catenin binding, neurogenesis, histone methyltransferase binding, central nervous system development, and protein dimerization activity (Figure 6(a)). In addition, the high expression level of MMP9 is associated with signaling receptor binding, lipid binding, collagen trimer, transition metal ion binding, calcium ion binding, and midbody. The low expression level of MMP9 is associated with beta-catenin binding, structural molecule activity, nuclear body, protein dimerization activity, and cytoskeleton organization (Figure 6(b)). In terms of SPI1, the high expression is correlated with negative regulation of gene expression, cell cycle, BORC complex, and smoothened signaling pathway, while the low expression is correlated with the phosphatase binding, transcription regulator activity, chromatin binding, misfolded protein binding, synapse, and structural molecule activity (Figure 6(c)). Finally, the protein dimerization activity, transcription regulator activity, structural molecule activity, nuclear body, and toxic substance binding are associated with the high expression level of VEGFA. However, the low expression level of VEGFA is closely associated with the pathways, such as transition metal ion binding, signaling receptor binding, midbody, lipid binding, and intrinsic component of the plasma membrane (Figure 6(d)).

## 4. Discussion

Over 27 million Americans are estimated to have osteoarthritis (OA), also known as degenerative joint disease. Degenerative diseases can affect any joint [16]. Articular cartilage and surrounding tissues are primarily affected by OA. OA can be classified as either primary or secondary. In primary OA, the disease is idiopathic, usually affecting multiple joints at the same time and usually affecting those who are relatively elderly. In most cases, secondary osteoarthritis is a single-joint condition caused by an articular surface disorder, such as trauma [17]. In recent years, although many researchers have put a lot of effort to explore the potential mechanisms of OA, little progress has been achieved. Therefore, it is urgent to explore the potential biomarkers for better diagnosis, diagnosis, and prognosis of OA. In addition, with the development of bioinformatics analysis, many studies focused on the use of bioinformatics analysis in various diseases. In this work, by combining the traditional RNA sequencing analysis and the single-cell RNA sequencing analysis, we aim to explore the genes that play a key role in OA. Firstly, we performed the differentially expressed analysis to explore the genes that play a key role in the OA patients. In addition, based on the DEGs, we also performed pathway enrichment analysis. The results demonstrated that humoral immune response, complemental and coagulation cascades, and regulation of inflammatory response are the most enriched pathways. The presence of OA is associated with low-grade inflammation. OA patients frequently experience synovial inflammation due to macrophages, T cells, B cells, and other immune cell infiltration, which plays an important role in its pathogenesis [18]. As a result, it is extremely important to coordinate the local inflammation microenvironment with the regeneration microenvironment during OA treatment [19]. It has been shown that GRB10 and E2F3 can be used as diagnostic markers of osteoarthritis, and they are important in the occurrence and development of this condition [20].

Subsequently, to further explore the immune-related genes that play a key role in the OA, we performed the PPI network analysis. The results revealed that some genes showed many interactive counts with other genes, which were regarded as the hub genes, such as MMP9, VEGFA, SPI1, IRF8, and CAMP. The single-cell RNA sequencing analysis was also applied to evaluate the specific expression of hub genes in specific cells. The results demonstrated that osteoclast is the most enriched cell in the synovial tissues of IRF8. It has been found that the degeneration of chondrocytes and loss of subchondral bone can be prevented and treated by inhibiting the degeneration of chondrocytes. Other studies demonstrated that osteoclasts play a protective role in the prevention of OA by attenuating the loss of preexisting cartilage through osteoclast-mediated bone loss. Therefore, the osteoclast plays a key role in the process of OA. In addition, many studies focused on the role of VEGFA in OA. The process of angiogenesis, or the formation of new blood vessels, contributes significantly to the pathogenesis of a wide range of human diseases. As a heparin-binding growth factor, vascular endothelial growth factor A (VEGFA) is among the known angiogenic factors [21]. Angiogenesis, migration, proliferation, and the formation of oviducts are all mediated by VEGFA, a tyrosine kinase glycoprotein [22]. Furthermore, it is involved in skeletal development, osteoblasts, and osteoclasts, which are involved in endochondral bone formation by coupling angiogenesis with hypertrophic cartilage remodeling [23]. Inflammation and angiogenesis are important pathological changes of KOA cartilage; degeneration of the cartilage is time-dependent. According to former study, the expression of the VEGFA/VEGFR2 pathway was mainly affected by KOA levels [24]. In this work, the single-cell RNA sequencing analysis demonstrated that progenitor cell, stem cell, osteocyte, osteoblast, osteoclast, and mesenchymal stem cell are closely associated with the VEGFA. Also, we also explore the potential pathways that are closely associated with VEGFA. The protein dimerization activity, transcription regulator activity, structural molecule activity, nuclear body, and toxic substance binding are associated with the high expression level of VEGFA.

In spite of the fact that secondary analysis of online data can provide many exciting results [25, 26], as far as our understanding of underlying disease mechanisms goes, this progress has been less dramatic than expected, especially when comparing genotypes and phenotypes [27]. In spite of the progressive increase in the number of key genes available through network-available online databases, significant limitations remain in the translation of these valuable data into disease-focused explanations [28]. It may be necessary to combine traditional analytical approaches with strategies aimed at interrogating complex biological systems in order to achieve this goal [29].

In conclusion, VEGFA is considered as the key immune-related gene that may play a key role in patients with OA. In addition, the single-cell RNA sequencing analysis revealed that osteoclasts also play a key role in the process of OA.

## Data Availability

The data used to support the findings of this study are included within the article.
